# Semantic Extraction of Permanent Structures for the Reconstruction of Building Interiors from Point Clouds

**DOI:** 10.3390/s20236916

**Published:** 2020-12-03

**Authors:** Inge Coudron, Steven Puttemans, Toon Goedemé, Patrick Vandewalle

**Affiliations:** 1 Flanders Make, 3001 Heverlee, Belgium; inge.coudron@kuleuven.be; 2 Flanders Innovation & Entrepreneurship (VLAIO), 1030 Brussel, Belgium; steven.puttemans@vlaio.be; 3 EAVISE, PSI, Department of Electrical Engineering (ESAT), KU Leuven, Jan Pieter De Nayerlaan 5, 2860 Sint-Katelijne-Waver, Belgium; toon.goedeme@kuleuven.be

**Keywords:** deep learning, semantic segmentation, semantic completion, indoor 3D reconstruction

## Abstract

The extraction of permanent structures (such as walls, floors, and ceilings) is an important step in the reconstruction of building interiors from point clouds. These permanent structures are, in general, assumed to be planar. However, point clouds from building interiors often also contain clutter with planar surfaces such as furniture, cabinets, etc. Hence, not all planar surfaces that are extracted belong to permanent structures. This is undesirable as it can result in geometric errors in the reconstruction. Therefore, it is important that reconstruction methods can correctly detect and extract all permanent structures even in the presence of such clutter. We propose to perform semantic scene completion using deep learning, prior to the extraction of permanent structures to improve the reconstruction results. For this, we started from the ScanComplete network proposed by Dai et al. We adapted the network to use a different input representation to eliminate the need for scanning trajectory information as this is not always available. Furthermore, we optimized the architecture to make inference and training significantly faster. To further improve the results of the network, we created a more realistic dataset based on real-life scans from building interiors. The experimental results show that our approach significantly improves the extraction of the permanent structures from both synthetically generated as well as real-life point clouds, thereby improving the overall reconstruction results.

## 1. Introduction

Three-dimensional (3D) modeling of building interiors has attracted increased attention in recent years due to the growing demand for realistic 3D models in a wide range of virtual and augmented reality applications. These include localization and navigation in public buildings, building construction monitoring or virtual tours in buildings. For newer buildings, a 3D building information model (BIM) usually already exists, but for older buildings, this is not always the case. When a building information model is not yet available, one has to be constructed from imagery and/or point clouds. A building interior model is often still created manually. However, this is very time-consuming and requires expert knowledge of 3D software tools. Automatic approaches can make the process easier, faster and cheaper.

**Figure 1:**
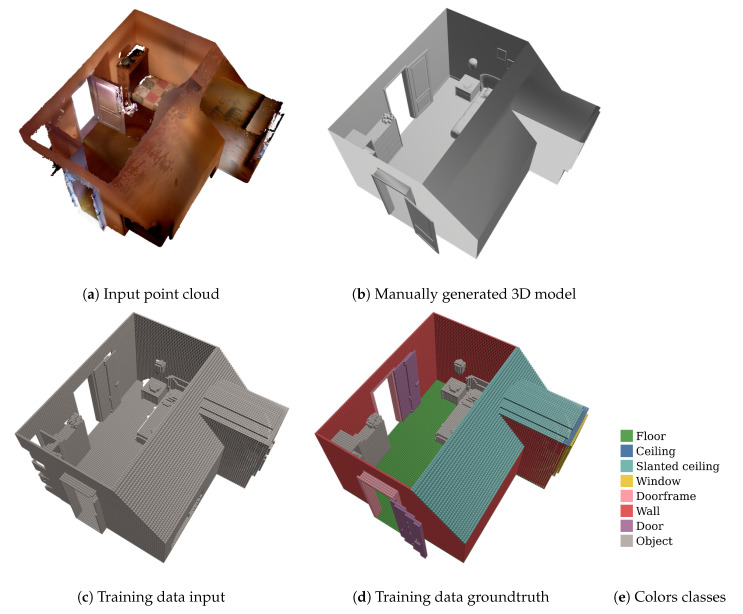
Overview of the extraction of our synthetic training data.

In fact, a wide range of approaches exists for automatically modeling building interiors from point clouds. Obtaining a 3D interior model has been found quite challenging due to the presence of noise, occlusion, and clutter [1,2,3,4,5,6,7,8,9,10]. An important step in any indoor scene reconstruction method is the extraction of permanent structures (such as walls, floors, and ceilings) as they define the layout of the rooms that have to be reconstructed from the scanned point cloud. These permanent structures are, in general, assumed to be planar. However, as the scanned point cloud also contains other objects, such as furniture, cabinets, not all planar surfaces that are extracted belong to permanent structures (see Figure 1a). As a result, geometric errors in the reconstruction, such as indentations near closets or beds, can occur when planar surfaces of these closets or beds have been wrongly extracted as permanent structures. Therefore, it is important that indoor scene reconstruction methods can correctly detect and extract all permanent structures even in the presence of clutter.

In [1], the authors try to minimize the number of falsely positive detected permanent structures by applying graph-based reasoning. Permanent structures that are found to be adjacent are connected by an edge. Only permanent structures belonging to a valid structural path within the graph are extracted. This approach itself was not validated in their paper, but it was mentioned that not all permanent structures were correctly identified. Therefore, they allowed manual corrections to the detection of permanent structures, making the approach semi-automatic. Another semi-automatic approach to as-built building information modeling was proposed by Hong et al. [7]. They create a 3D wireframe using horizontal and vertical modeling restrictions, and use this as input to a manual refinement step.

Jung et al. designed an automated reconstruction method for multiple-room building interiors using explicit modeling of floors and walls, and applied a regularization using Manhattan world assumptions [8]. Tran and Khoshelham presented a stochastic approach for reconstruction from point clouds, using reversible jump Markov chain Monte Carlo sampling (rjMCMC) [6]. Their algorithm was tested both on Manhattan and non-Manhattan structures. A detailed comparison between point clouds and meshes for segmentation and classification of building interior components was performed by Bassier et al. [9]. They focused on manually designed features, whereas we will focus in this work on deep learning approaches, where the features are also learned automatically. Ma et al. developed a method to extend a small dataset of captured data with synthetically generated point clouds from BIM models [10]. Using this approach, they improved results with deep learning methods for semantic segmentation.

In this work, we propose to perform semantic scene completion using deep learning for the automatic extraction of permanent structures. We started from the ScanComplete network proposed by [11]. However, this network assumes that prior knowledge about the scanning trajectory is available, which is not always the case. Furthermore, the SUNCG dataset [12] on which the network was trained did not represent real-life scanned data very well. The work presented in this paper includes the following contributions:

- An optimization of the baseline architecture to reduce inference and training time. To achieve this, we removed some unnecessary operations and performed loop unrolling.
- An adaptation of the baseline architecture such that no information about the scanning trajectory is required. Instead of a truncated signed distance field data representation, we proposed a Manhattan distance based data representation.
- A more realistic synthetic dataset based on scans of building interiors from real-life houses.
- An ablation study on several design choices. In one of the experiments, we show that using previous voxel group predictions instead of ground truth values for training improves the overall results.
- A case study in which we use our adapted and optimized architecture to improve the extraction of planar permanent structures in challenging cluttered indoor environments.

The results presented in this paper show the synergy and combined effect of these contributions, while it is not always easy to show them in isolation due to their mutual interactions. The remainder of this paper is organized as follows: Section 2 describes the related work. The neural network and its adaptation is presented in Section 3, as well as the dataset we constructed. The experimental results are presented in Section 4, which is followed by our conclusions in Section 5.

## 2. Related Work

As explained previously, the goal of this work is to incorporate deep learning into the pipeline for the extraction of permanent structures from point clouds. This can make interior modeling methods more robust to occlusions and clutter. As clutter may lead to falsely positive detected permanent structures, the information from the semantic segmentation can be used to eliminate these false positives. Occlusions due to clutter might also cause permanent structures to remain undetected. In this case, scene completion can provide a way to detect these missing permanent structures. Therefore, we need semantic segmentation as well as scene completion to make the reconstruction pipeline more robust. We will briefly review related work on both scene completion and semantic segmentation.

### 2.1. Semantic Segmentation

Semantic segmentation has already been studied for a long time, first in 2D and more recently in 3D as well. Similar to assigning a semantic label to each pixel in an image, 3D semantic segmentation assigns a semantic label to each 3D point in a point cloud or to each voxel in a voxel grid, depending on the input representation. Before deep learning was widely adopted, traditional feature extraction approaches such as [13] were applied for the task of semantic segmentation. However, through the recent advances in deep learning, approaches based on deep learning have found their way into 3D semantic segmentation. Depending on the input representation, these approaches can roughly be split into two groups: methods that impose a regular grid on the unstructured 3D point cloud (by projection or voxelization), and methods that operate directly on the 3D point cloud.

One way to impose a regular grid on an unstructured point cloud is by converting the point cloud to an occupancy grid. The network architecture can then employ 3D convolutions to yield voxel-level predictions [14,15,16]. However, voxel grid representations are often memory inefficient due to the sparsity of 3D data as the number of unoccupied voxels is relatively large. Besides, as 3D convolutions are computationally more expensive than their 2D counterparts, the voxel size is often either constrained to a relatively large size or the spatial extent of the voxel grid is limited. It is, therefore, challenging to extend these methods to large-scale point clouds. To solve this problem, ref [17] proposed to use octrees instead. SEGCloud [18] on the other hand subsampled the large cloud into voxels and used trilinear interpolation and conditional random fields to obtain point-level predictions. To avoid training on the 3D data directly, indirect segmentation methods have been proposed. Lawin et al. [19] project a 3D point cloud onto a set of synthetic 2D images, which are then used to predict semantic labels of the projected points using a 2D convolutional neural network (CNN). However, the mapping from a sparse representation to a dense one leads to an increased memory footprint as well.

Alternatively, a new set of methods has been developed that can segment irregular data representations. PointNet [20] was the first network to operate directly on 3D point clouds. The architecture is composed of two subnetworks: one for classification and another one for segmentation. The classification subnetwork generates point features through a sequence of multi-layer perceptrons for each point individually, followed by a max-pooling layer to generate a global feature that describes the original input. The segmentation subnetwork then concatenates the global feature with the per-point features extracted by the classification network and applies another sequence of MLPs to produce the final output scores for each point. Many future works followed this approach and have also demonstrated excellent performance on the semantic segmentation task [21].

### 2.2. Shape Completion

Raw point cloud data is often sparse and incomplete. As a result, several methods have been proposed to produce a full 3D output from the sparse and incomplete input data. This task is typically called shape completion. Traditionally, these methods focused on filling small holes by fitting geometric primitives, or through continuous energy minimization [22,23,24]. Other approaches have been suggested that use shape priors or symmetry to complete the missing surfaces [25,26,27,28]. Although these methods show promising results, they are not able to generalize well or handle more complex scenarios.

To generalize to arbitrary new shapes, data-driven approaches using machine learning techniques have been proposed. The vast majority of these approaches use occupancy grids as data representations. Voxlets [29], for example, trained a random decision forest to predict unknown neighboring voxels. The final mesh was then constructed by averaging the prediction results and performing a marching cubes approach. One of the first approaches to use 3D convolutions was 3D ShapeNets [30], which learns a probabilistic distribution for 3D reconstruction from partial input shapes with a convolutional deep belief network. Since then, various other methods using 3D CNNs have been proposed [31,32]. While earlier 3D CNN approaches leveraged occupancy-based voxel representations, more recently other representations have been employed as well, such as discrete signed distance field (SDF) voxel grids, depth maps or point clouds [33,34].

### 2.3. Semantic Scene Completion

Semantic scene completion is the combined task of semantic segmentation and scene completion. SSCNet [12] was the first one to combine the two tasks and showed that completion and segmentation can actually benefit from each other. However, SSCNet takes a single depth image as input and therefore does not take advantage of the semantic features that can be learned from an RGB image. Afterwards, approaches such as [35,36] have added RGB features into the network. The See And Think network proposed in [37] for example, first performs a 2D semantic segmentation on the RGB-D input image and then performs 3D convolutions on the voxel grid obtained by back-projecting the image coordinates.

As the 3D reconstruction from densely overlapping RGB-D images is a relatively more complex problem due to the larger memory requirements, the aforementioned approaches all focus on scene reconstruction from a single image or a small set of RGB-D images. However, recently ScanComplete [11] has been proposed as the first approach for data-driven scan completion of large-scale 3D scenes. By leveraging a fully convolutional, auto-regressive approach at multiple subsequent resolutions, the method is able to predict complete geometry along with 3D semantic segmentation for complete 3D scenes. These characteristics make the ScanComplete network architecture most suited for improving the extraction pipeline of permanent structures. Hence, we choose it as a starting point for our proposed approach.

## 3. The Proposed Approach

In this section, we explain in detail the proposed multi-scale aggregation architecture for semantic scene completion. First, we give an overview of the ScanComplete network (the baseline network architecture). Next, we present our proposed adaptations, including the optimized network architecture and the strategy we used to eliminate the need of scanning trajectory information by changing the input data representation. After that, we describe how we generated our custom synthetic dataset to train the network. Finally, we explain how the output of the network, which consists of the semantically segmented and completed point cloud, can serve as input for a more robust extraction of permanent structures from the point cloud.

### 3.1. Baseline: ScanComplete Network

The ScanComplete network [11] predicts a complete 3D model along with per-voxel semantic labels from an incomplete 3D scan adopting a coarse-to-fine strategy. The partial 3D input scan is encoded as a truncated signed distance field (TSDF) in a volumetric grid. Positive values indicate voxels in front of the object [38]. As a result, the sign encodes which voxels are known and which are unknown. The principal output of the network is the truncated distance field (TDF). In contrast to the input, the output has no sign as the 3D models from which the synthetic dataset is generated are rarely watertight. Besides, as all voxels in the output are expected to be known, the sign is indeed no longer relevant.

By using this type of dense grid representation, the memory becomes a bottleneck. That is, the memory required to store grid representations grows cubically with the spatial extent. Hence, as the amount of memory available is limited, the network can either process a large scene using a low resolution or process a small scene using a high resolution. The disadvantage of the former is, however, that local detail is lost. The disadvantage of the latter is that, due to the smaller spatial extent of the scene, the scene might not provide enough context to perform accurate segmentation and completion. To overcome these drawbacks, the network adopts a coarse-to-fine strategy.

**Figure 2:**
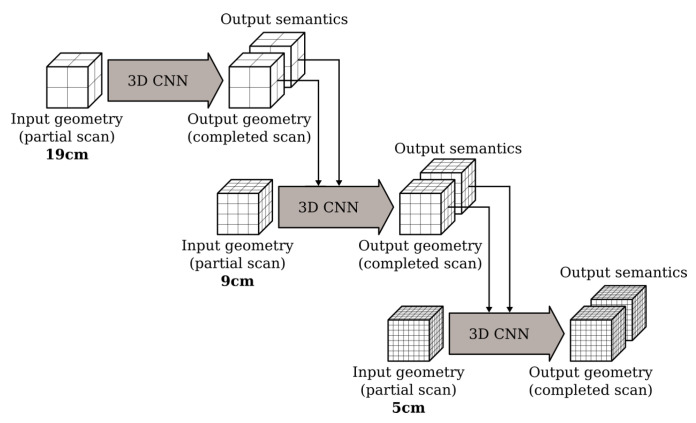
Overview of the hierarchical coarse-to-fine strategy.

Starting with a low (i.e., coarse) resolution, the network can process a larger spatial extent that provides more global information about the input. The results from this step are then used by the subsequent hierarchy that operates on a higher (i.e., finer) resolution to produce more fine-grained predictions. The ScanComplete network architecture operates on 3 subsequent grid resolutions. From coarse to fine it processes the input at 19 cm, 9 cm and 5 cm resolution as shown in Figure 2. Note that the network architecture is not limited to these hierarchy levels. The number of hierarchy levels as well as their resolution can be changed.

**Figure 3:**
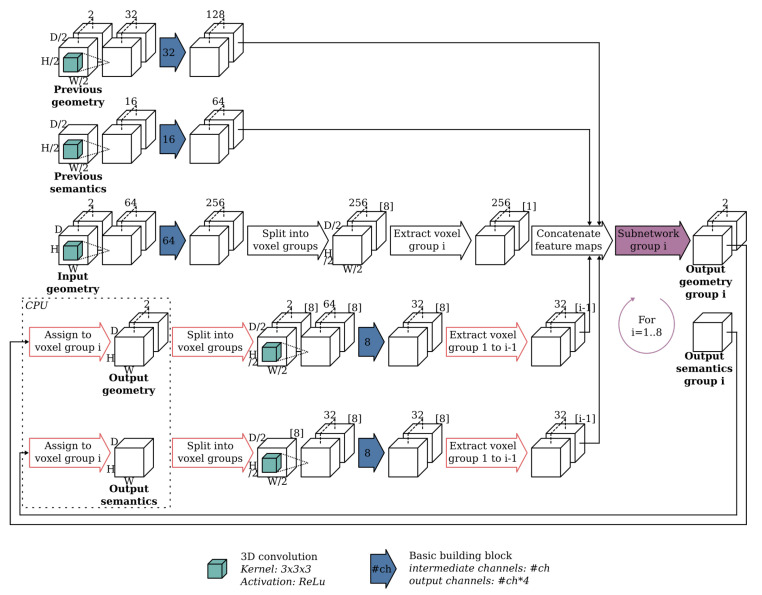
Overview of the baseline 3D CNN for a single hierarchy. Note that for the first hierarchy (19 cm) there are no previous predictions. The blue arrows represent the basic building block as shown in Figure 4. For each basic building block, the parameter value is displayed that represents the number of channels used by the 3D convolutions from the basic building block.

**Figure 4:**
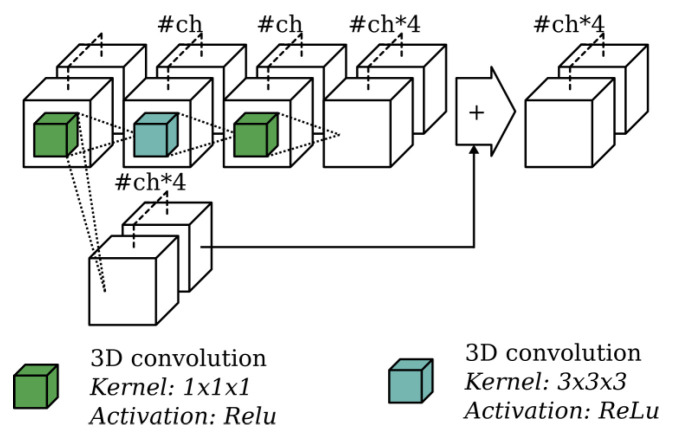
Overview of the basic building block used in ScanComplete where #ch is a parameter value describing the number of channels used for the 3D convolutions and #in represents the number of input channels of the first convolution.

At each resolution, the network uses an autoregressive architecture similar to the one presented by [39]. The key idea behind their network is to speed up the conditional image generation proposed in pixelCNN [40] by dividing the pixels into disjoint groups such that pixels from the same group can be sampled in parallel. The ScanComplete network extends this idea to the third dimension by dividing the voxels in the voxel grid into 8 groups such that voxels from the same group do not neighbor each other, as shown in Figure 2 of [11]. Each group of voxels is then generated autoregressively by conditioning on the predictions of the groups that precede it as shown in Figure 3 (making use of the building block shown in Figure 4). As a result, 8 separate subnetworks must be learned, one for each voxel group at each hierarchy level (3 in total). Hence, in total 24 subnetworks must be learned.

**Figure 5:**
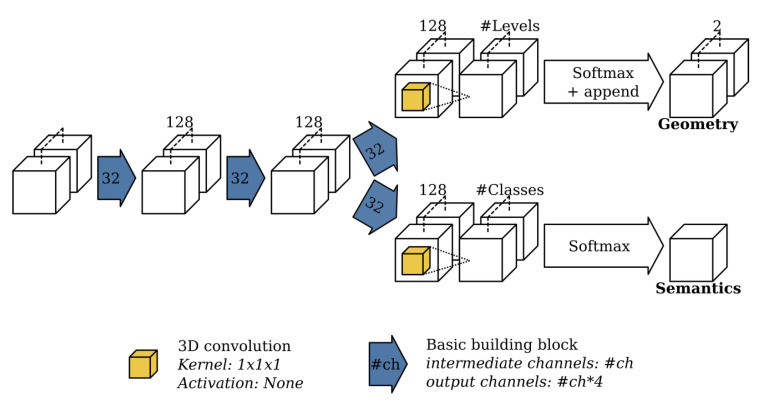
Overview of the subnetwork used to predict each group. The blue arrows represent the basic building block as shown in Figure 4. For each basic building block, the parameter value is displayed that represents the number of channels used by the 3D convolutions from the basic building block.

The subnetworks consist of 3D convolutions together with 1×1×1 shortcuts as shown in Figure 5. The previous voxel groups on which the current voxel group is predicted, are concatenated feature-wise. At the end of each subnetwork, the final prediction is split into two parts. The first outputs the completed TDF voxel grid, while the second consists of a voxel grid with semantic labels per voxel. Finally, all voxel group predictions must be reassembled into a voxel grid with the same spatial extent as the input scene. Note that since the network is fully convolutional, it can be trained on random spatial crops from training scenes, but tested on scenes of arbitrary spatial extent.

### 3.2. Overview of the Optimized Architecture

The baseline ScanComplete network as implemented by [11] was extremely slow and could, therefore, not be used in practice. The main reason for this is that in Tensorflow 1 it was not possible to assign a new value to a variable in the computational graph during graph execution. However, as explained previously the network autoregressively computes each voxel group conditioned on the predictions of the previous voxel groups. Hence, the output predictions of a subnetwork are needed by each of the subsequent subnetworks. Dai et al. [11] implemented this by subsequently replacing the voxel group from the variable that holds the output prediction with the computed predictions of that voxel group for each subnetwork in the loop. However, as this assignment operation cannot be done within the computational graph, the variable output prediction was stored outside the graph in CPU memory. Hence, for each subnetwork this variable had to be reloaded into GPU memory. Furthermore, the baseline implementation made extensive use of splitting and extraction operators (slicing), which are considerably more expensive in Tensorflow [41]. Additionally, the feature maps for the previously predicted voxel groups were recalculated for each voxel group by each subnetwork even though they were already calculated by the previous subnetworks. This resulted in an additional and unnecessary overhead.

**Figure 6:**
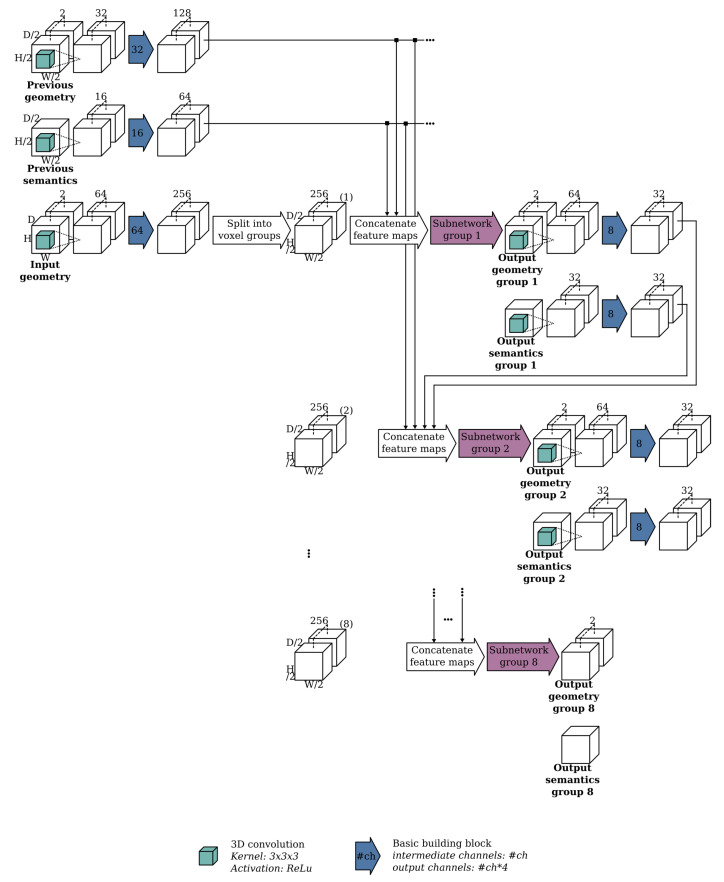
Overview of our optimized architecture of the 3D CNN for a single hierarchy. The blue arrows represent the basic building block as shown in Figure 4. For each basic building block, the parameter value is displayed that represents the number of channels used by the 3D convolutions from the basic building block.

To overcome these issues, we unrolled the for loop that is inherently present in the baseline implementation as shown in Figure 3. The intermediate output predictions of each subnetwork were kept in GPU memory. Only after all 8 subnetworks have been processed, the final output prediction was reassembled. As a result, the assignment and extraction operators, in the red arrows in Figure 3 could be removed as they were no longer necessary. Furthermore, the loop unrolling allowed to reuse the feature maps for each predicted voxel group and concatenate them to the input feature maps of each of the subsequent voxel groups. Hence, this allowed us to reduce the processing time by approximately a factor 10. See Figure 6 for an overview of our processing architecture. While the overall architecture remains the same, the optimization also significantly improves the segmentation performance as shown in Table 1 of the ablation study. The loop unrolling allows to train all 8 subnetworks end-to-end, in contrast to the original training procedure from [11], in which each subnetwork was trained conditioned on the previous voxel groups from the ground truth data.

### 3.3. Overview of the Proposed Input Data Representation

In the baseline ScanComplete network, the input is encoded as a truncated signed distance field (TSDF) in a volumetric grid. An approximation of this distance field is often computed as the distance to the nearest object point on the line of sight from the camera, meaning that the camera pose must be known. This is also called the projective truncated signed distance. However, as mentioned before, we want to eliminate this view dependency so that we can process raw point clouds as well.

To do so, the authors from [12] suggested calculating the truncated signed distance field using another method which is slower but more accurate. Instead of only considering the closest point in the line of sight of the camera, the distance is computed to the closest point anywhere on the reconstructed surface. This implies that first a surface is reconstructed from the point cloud. However, most surface reconstruction algorithms require either oriented normals or camera viewpoint information. As we will not have viewpoint information available, we must recover the correct orientation of the normals in the point cloud alone otherwise the reconstruction will lead to spurious geometric or topological distortions. Finding a consistent orientation of the normals is, in general, an ambiguous problem. Then again, if the point cloud is acquired from a single depth image, as is the case in [12], this ambiguity is easily solved by the fact that the normals should point towards the camera that observes them. As a result, the approach suggested in [12] is in fact not truly view-independent and cannot be used in our case.

Alternatively, the point cloud can be converted into a binary occupancy grid. However, according to [42], a binary-occupancy grid representation is not suitable for training. Due to the sparsity of this representation, the gradient flow during training is not fluent, making it difficult for the network to learn. Therefore, we propose to convert the point cloud into a volumetric distance field based on the $L_{1}$ distance or Manhattan distance. For each voxel, the Manhattan distance to the nearest occupied voxel is computed. To avoid boundary effects when zero-padding the volume, the distance values are flipped and truncated:

(1) $$d=\max\left\{d_{max}-{∥x-x_{o}∥}_{1},0\right\}$$

where *d* is the distance value at voxel x=(i,j,k) and $x_{o}=\left(i_{o},j_{o},k_{o}\right)$ represents the nearest occupied voxel. The $L_{1}$ distance or Manhattan distance is calculated as

(2) $${∥x-x_{o}∥}_{1}=|i-i_{o}|+|j-j_{o}|+|k-k_{o}|.$$

The benefit of using the Manhattan distance field is two-fold. Firstly, the volumetric representation becomes less sparse compared to a binary representation. Secondly, the Manhattan distance is also more robust. If the distance field would be computed using a euclidean distance from the voxel center to the nearest point in the point cloud, the distance would be much more susceptible to noise and point density.

Additionally, we propose to add an extra channel based on the results from [12]. In that paper, different surface encodings were explored. Experiments showed that the accurate TSDF has less view dependency than the projective TSDF. However, the accurate TSDF still suffers from strong gradients along the boundary between the known and unknown space. Therefore, they suggest flipping the truncated signed distance values, so that the largest gradient is on the reconstructed surface. This provides a more meaningful signal for the network to learn geometric completion. To ensure we have a large gradient near the surface as well, we add an extra channel with the following encoding:

(3) $$s=\left\{\begin{array}{cc} 1 & \mathrm{if}\;d=d_{max}\\ -1 & \mathrm{if}\;d=d_{max}-1\\ 0 & \mathrm{else}. \end{array}\right.$$

For future reference, we will call our Manhattan-based distance field the flipped truncated distance field or FTDF.

### 3.4. Training Data Generation

To train the ScanComplete network, we build our own dataset. The primary reason for this is that we had to adapt the training data to suit our needs, which deviate from the setup used in the ScanComplete work in several aspects. Firstly, the input data representation from the baseline ScanComplete network requires the availability of the depth frames together with the camera poses between them in order to be able to fuse the depth data into a truncated signed distance function volume. However, this information is not always available depending on the device that was used to create the scan. Therefore, we explored a different input data representation as explained previously.

**Figure 7:**
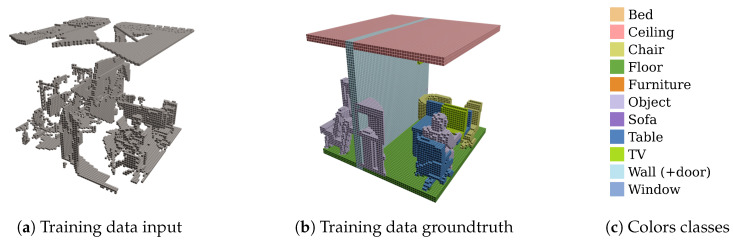
Excerpt of the training data in the SUNCG dataset.

Secondly, as opposed to the SUNCG dataset, our input data consists of single rooms being processed one at a time instead of complete floor levels. As the spatial context is different for a single room than for a complete floor level, the features the network will learn will differ. For example, walls between adjacent rooms result in pairs of parallel surfaces. This information can be exploited by the network to learn how to distinguish between walls and other objects such as cabinets. For a single room, each wall marks the outer boundary of the room. As a consequence, we created a synthetic dataset that consists of single rooms, such that the training data better resembles the actual input data. An excerpt from the training data in the SUNCG dataset is shown in Figure 7.

Furthermore, the semantic labeling that was used in the SUNCG dataset did not fit our requirements. For example, the SUNCG dataset has one combined class for walls and doors. However, in our use case, it is important that these two categories are split into different classes. The main objective of using this network is to provide a more robust extraction of permanent structures for the reconstruction of building interiors. As open doors must not be seen as permanent structures that define the interior geometry of the room, separate classes are required to be able to distinguish between them. Changing these labels manually would be extremely time-consuming as the dataset contains over 200,000 spatial crops.

Finally, the complexity of the room geometries available in the SUNCG dataset is very limited. Firstly, the height of the rooms is the same for all rooms in this dataset. Secondly, the geometry is restricted to straight walls and ceilings. However, rooms on the top floor of a house often have dormers and sloped ceilings, which are not present in the SUNCG dataset. Therefore, it was necessary to build our own dataset, as the SUNCG dataset does not fit our needs and does not represent the real world very well either.

**Figure 8:**
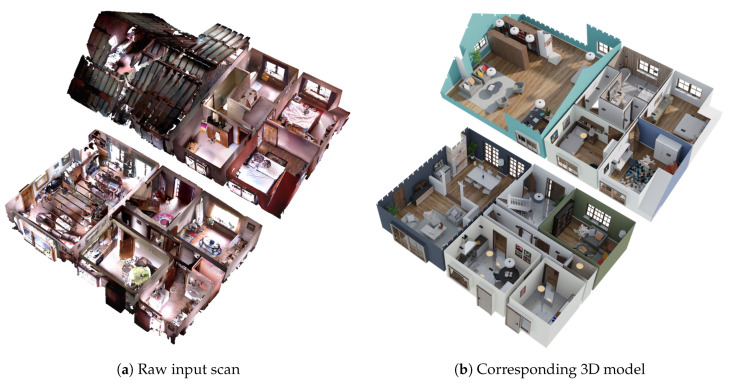
Example of a raw input scan together with the corresponding 3D model created by a Blender artist.

To build our own dataset we start from 3D interior scans of houses that were made using a consumer handheld 3D scanner (e.g., DotProduct). Obtaining a completed and annotated version of the scans for training is however not feasible as it would require manually completing and annotating the point clouds from the scans. Therefore, a synthetic dataset was created based on this real dataset. The raw pointclouds from the real dataset were given to external parties (freelance Blender artists) specialized in 3D modeling of houses, who used the 3D modelling tool Blender [43] to create a mesh model of the room layout from these point clouds. These mesh models of the room layouts follow the structure of the room as closely as possible. Afterwards, this mesh model is used as a base layer in a visualization tool, which allows you to decorate and furnish the house. In Figure 8, we can see an example of the final decorated and furnished mesh model that was manually created based on the raw point cloud data obtained from the 3D scanner.

From these mesh models, we then generated our synthetic dataset. As the input data for our deep learning architecture consists of an incomplete 3D scan, we must first create a 3D scan from the mesh model. Different approaches exist to obtain a 3D scan from mesh models by creating an artificial scanning trajectory as proposed for example by [11] or [44]. However, to make the scanning trajectory more realistic, we reused the scanning trajectory from the scans of the real dataset. Although the data format of the scans from the 3D scanner was closed source, a plugin for CloudCompare [45] was available to load the scans, making it possible to extract the scanning trajectory from the scans. The camera viewpoints from the scanning trajectory were then used to render depth images from the mesh model. These depth images were then transformed back into 3D point clouds and from there into the volumetric representation.

Next, to generate the ground-truth output data, the mesh models are first converted to point clouds by sampling points over the surface of the mesh models. The point clouds are then transformed into the volumetric representation as described previously. The freelance Blender artists were also asked to consistently label each part or object within the mesh model during creation of the mesh model. The color of the points in the point cloud corresponds to the class label given by the freelance Blender artists to the mesh part from which the points were sampled. The class labels of the point cloud are transferred to the voxel space by means of a priority vote. As each class was given a priority, the voxel is assigned the class label of the point with the highest priority. The classes are listed in order of priority in Figure 1e with the highest priority class at the top.

Note that each record in the dataset consists of a volumetric representation of the input scan and the corresponding labeled output scan of a single room. Therefore, the size of the volumetric grids varies from one record to another. To train a convolutional neural network on variable-size input data, a number of data preparation paths can be taken. One approach would be to extract fixed-size crops from the original input data. As another option, we can train the neural network with a batch size of 1. Alternatively, we can pad all records in a batch to match the size of the largest record in the batch. The effects of this decision will be discussed in Section 4.

### 3.5. Application: Semantic Extraction of Permanent Structures

**Figure 9:**
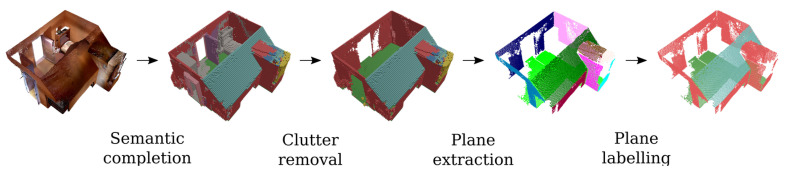
Overview of the semantic extraction step.

After the scanned point cloud has been labeled using the previously described deep learning framework, the clutter (i.e., voxels labeled as object, door or door frame) is removed from the point cloud. Then, the planar primitives representing the permanent structures are extracted from the point cloud. As most permanent structures are planar, we can extract planar primitives from the point cloud using methods such as RANSAC [46] or region growing [47]. Due to the discretization effect as a result of the voxelization, multiple almost coplanar planes were extracted representing the same permanent structure. Therefore, it was necessary to perform plane regularization [46]. Hence, nearly coplanar planes were made exactly coplanar using CGAL as well [46]. Finally, each plane was given a semantic label which can later be used by the semantic model of the room. For this, the majority vote was computed among the points that make up the plane. An overview of the semantic extraction step is shown in Figure 9.

## 4. Experimental Results

In this section, we first conduct an ablation study to analyze our design choices. As in [12], we split the evaluation results into two parts: scene completion and semantic scene completion. The scene completion only evaluates whether the voxels are correctly classified as occupied or empty by measuring the voxel-level intersection-over-union (IoU). The semantic scene completion, on the other hand, also evaluates whether the voxel is given the correct semantic label. The IoU is computed for each class separately as well as averaged across all classes. Next, we also compare our final architecture qualitatively with the baseline ScanComplete on our synthetic data. Finally, we also show some results of our approach on a real scan with an RGBD camera, showing the performance under noisy measurements.

### 4.1. Ablation Study

**Figure 10:**
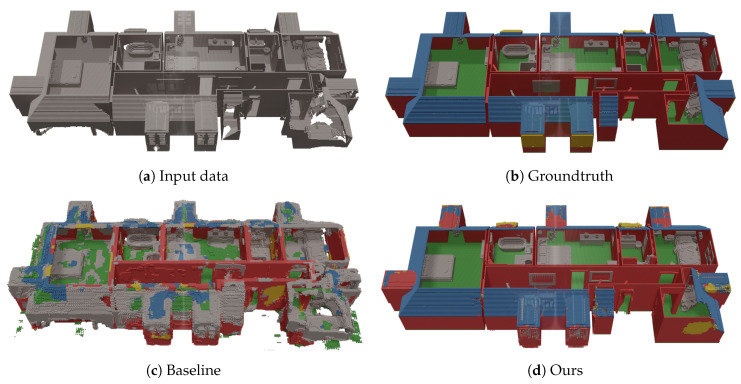
Qualitative evaluation of the baseline ScanComplete network compared to our implementation on a house from the test dataset (first floor).

**Figure 11:**
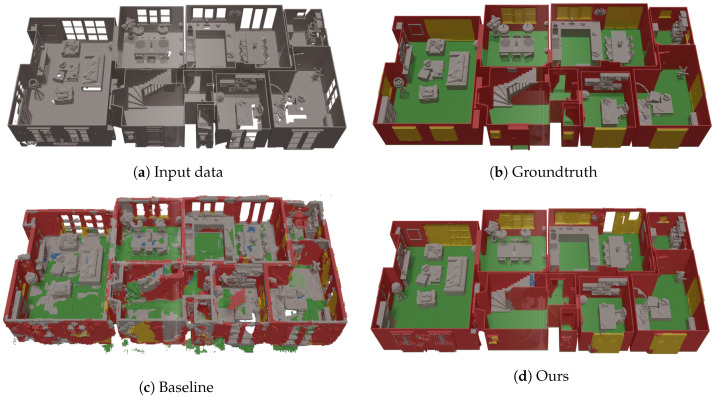
Qualitative evaluation of the baseline ScanComplete network compared to our implementation on a house from the test dataset (ground floor).

Table 1 and Table 2 summarize the quantitative results. All results from Table 1 were obtained by training and testing on (split parts of) the SUNCG dataset, while the results from Table 2 were obtained by training and testing on our dataset. In Figure 10 and Figure 11 a qualitative comparison between our network and the baseline ScanComplete network is shown. Using this framework, we will now evaluate a few research questions.

#### 4.1.1. Does Training on Previous Voxel Group Predictions Help?

As shown in Figure 6, a separate subnetwork is trained for each voxel group which is conditioned on the predictions from the previous voxel groups. In [11], the training of these subnetworks was sped up by feeding ground truth volumes as the previous voxel groups instead of the actual predictions. In this way, the subnetworks could be trained in parallel. However, in our experiments, this tends to actually result in larger misclassified regions. We hypothesize that this is because the network learns to rely heavily on the previous voxel groups having correct predictions. By training on previous voxel group predictions instead of ground truth volumes, the network can be trained end-to-end and must learn to cope with the uncertainty on the predictions of the previous voxel groups. Table 1 shows indeed that a large performance increase can be achieved in semantic scene completion by training conditioning on the previous voxel group predictions. The performance of the scene completion, on the other hand, did not change by training the network end-to-end.

#### 4.1.2. Can We Combine Multiple Object Classes?

In the SUNCG dataset, multiple object categories are defined such as table, chair, furniture or sofa. In our dataset, on the other hand, we only have a single object class that represents all these categories. Normally by reducing the number of classes, the performance of the network will increase. However, we wanted to verify if this is also the case for the ScanComplete network. The idea behind the network is that understanding the object geometry can help to recognize objects and vice versa. By combining all types of objects into a single class, the object class becomes very broad. Therefore, it might become more difficult for the network to learn how to recognize and complete all these objects. The results from Table 1 show however that it is still beneficial to combine these different categories into a single object class. In fact, this design choice has the greatest impact on the overall segmentation performance as the IoU increases from 30.90% for the baseline network to 58.84% with the combined classes.

#### 4.1.3. How Much Data Do We Need?

As our dataset is rather limited compared to the SUNCG dataset, we investigated how much the amount of data influences the performance. For this, we trained the network on a subset of our baseline dataset, containing only about 1% of the total number of records. The results from Table 1 show a significant drop in performance (26.61% with the limited data set vs. 30.90% for the full data set), suggesting that there will indeed be a significant performance gain if we continue to add new houses to our dataset.

#### 4.1.4. To Crop or to Pad?

Next, we investigated two data preparation strategies for handling variable-size inputs. To train a convolutional neural network on variable-size input data, we can either extract fixed-size crops from the original input data or we can pad all records in a batch to match the size of the largest record in the batch. In [11] the former strategy was used as their dataset consisted of complete floor levels. Due to memory limitations, it is not feasible to feed a complete floor level into the network. Therefore, they extracted random crops of 6 m × 3 m × 6 m (width× height × length) for the 19 cm resolution hierarchy and crops of 3 m × 3 m × 3 m for the 9 cm and 5 cm resolution hierarchy from each floor level as was shown in Figure 7. In our dataset, however, each record consists of a single room. Hence, instead of learning the relationship between rooms and objects in the room, the network must now learn to understand the outer boundary of a room. Therefore, extracting crops of this 6 m × 3 m × 6 m spatial extent might actually throw away a lot of otherwise useful information. A typical (Western-European) living room, for example, will be rectangular and will probably be larger than 6 m in one dimension. Furthermore, the height of the rooms might also exceed 3 m. Note that a drawback of padding is usually that it creates an artificial boundary around the input. This is however not the case in our dataset. For the geometry, a zero value simply means that the voxel is not occupied, while for segmentation a zero corresponds to an empty voxel. Consequently, we hypothesize that the network will perform better if the complete spatial extent is given as input when training the first, coarsest hierarchy. To improve efficiency we dynamically pad each room to match the dimensions of the largest room in the batch instead of the largest room in the dataset. The results from Table 2 show that for our dataset, the padding strategy indeed outperforms the cropping strategy as performed by Dai et al. Furthermore, by using the complete spatial extent of each room, the network becomes much better at predicting windows.

#### 4.1.5. Can We Remove the View Dependency by Changing the Input Encoding?

In this experiment, we compare how our proposed input encoding (i.e., FTDF) performs against the original input encoding from the baseline [11] (i.e., TSDF). We observe that removing the view dependency by using our proposed input encoding gives a 19.27% improvement in IoU. This is an important accomplishment as we wanted to be able to complete and segment raw point clouds that only contain XYZ data without losing performance.

#### 4.1.6. Does Data Augmentation Help?

As seen previously, having a larger dataset will improve performance. As our dataset is still limited, we try to artificially extend our dataset by applying data augmentations. To do this, we rotate each room by 90 degrees. Additionally, we can flip the rooms. As a result, the amount of data can be increased with a factor of 8. As shown in Table 2 this already improves performance from 64.54% to 69.52%.

### 4.2. Qualitative Evaluation of the ScanComplete Network

Finally, we select our best performing network and compare it qualitatively with the baseline ScanComplete network. As the datasets consist of a different set of classes, we aggregate the predictions into a common set of classes. In Figure 10 and Figure 11, we visualize the results of both networks on the synthetic test data. It is clear that the baseline ScanComplete network cannot handle our data very well. One reason for this might be the fact that we process each room separately instead of all floors from a single floor level at once. As a result, the baseline ScanComplete network is not able to complete our room-by-room input data.

Furthermore, we can see that the walls and floors have become much thicker than in the original input in case of the baseline ScanComplete network. This is a direct result of how the training data was made in the SUNCG dataset, on which the baseline ScanComplete network was trained, as shown in Figure 7. Finally, as expected, the baseline ScanComplete network cannot cope with the slanted ceilings and detects them as objects.

**Figure 12:**
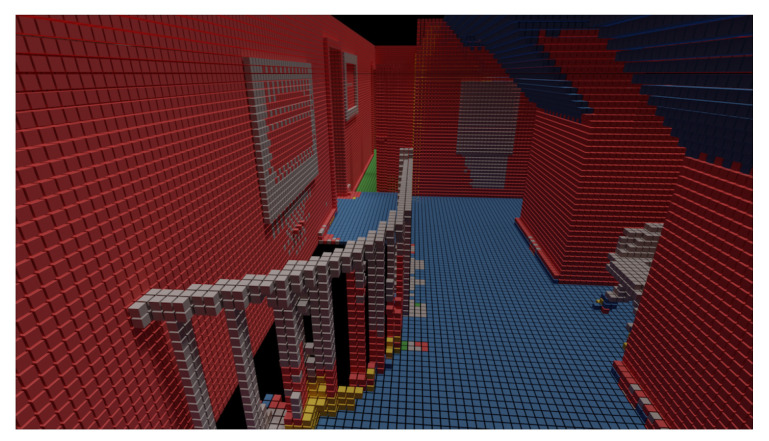
Floor level difference in hall has been wrongly labelled as ceiling.

However, there is one area where the baseline ScanComplete network and ours as well fail, as shown in Figure 12. There is in fact a small level difference in the floor of the hall on the first floor. Both parts should, of course, be labeled as floor, but the network labels only the lower part correctly as floor. The upper part of the floor is labeled is ceiling. We hypothesize that the reason for this is that this situation did not occur in our training data set as the data set is relatively small.

**Figure 13:**
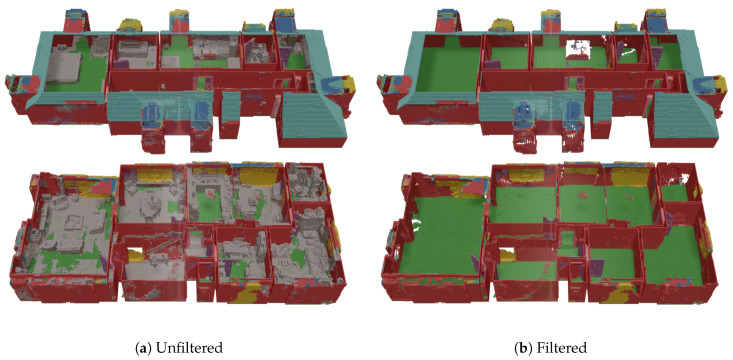
Qualitative evaluation of our implementation on raw input data.

Next, we look at the results of our approach on the raw input point cloud measured with a consumer RGBD camera. These are the real (noisy) measurement data, which were used as input for our manually created synthetic data set (applied in the rest of this paper). From the results in Figure 13, we can see that the results seem chaotic at first, especially on the ground floor. This is mostly due to the noisy character of the raw input cloud (captured with a consumer RGBD camera). As our synthetic input data is noise-free, the network did not learn this and labels the noise as objects. Hence, if we filter the labeled voxel grid and remove all voxels that were labeled as object, we can clearly see that the network is, in fact, able to segment and complete almost all planar permanent structures.

### 4.3. Semantic Extraction of Permanent Structures

**Figure 14:**
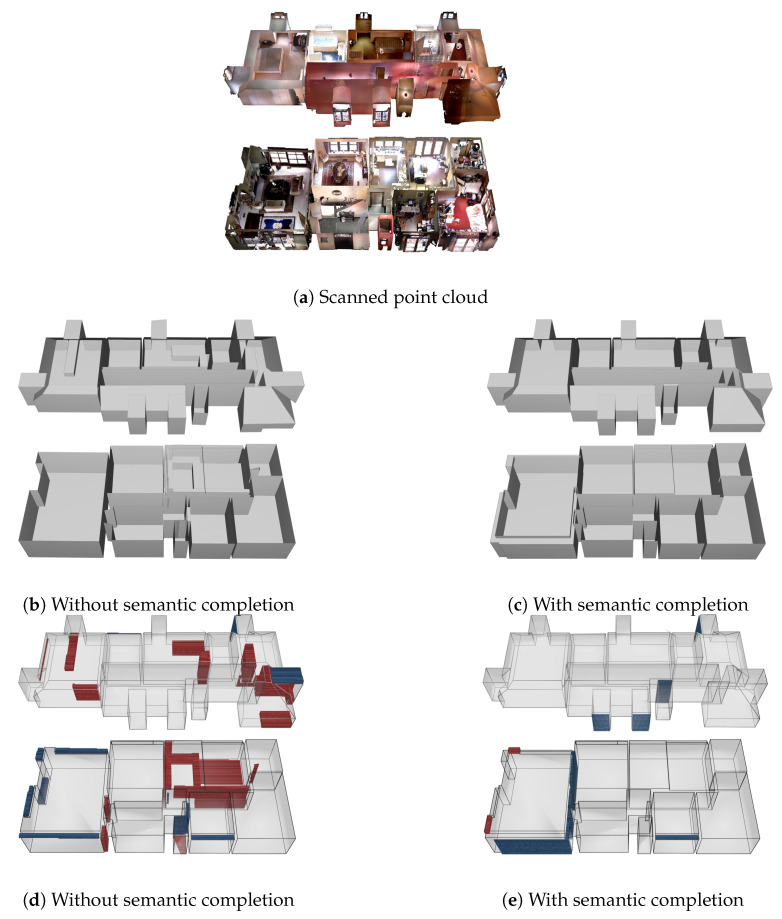
Qualitative comparison of the geometric reconstruction with and without semantic completion.

**Figure 15:**
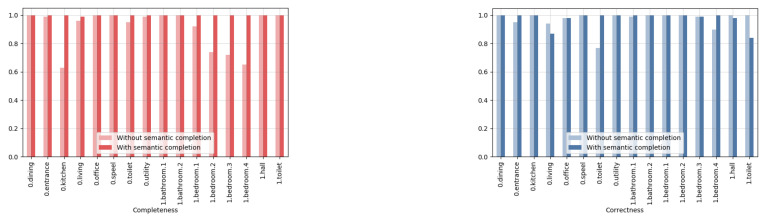
Quantitative comparison of the geometric reconstruction with and without semantic completion.

**Figure 16:**
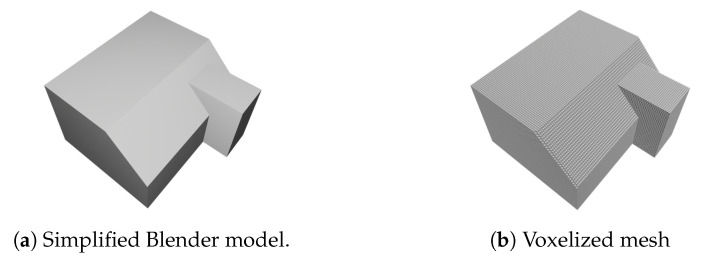
Visualization of the mesh voxelization.

In Figure 14, a qualitative comparison is shown between the reconstruction results with and without prior semantic completion. For the geometric reconstruction of the room layout from the extracted planar primitives, we used a similar pipeline as proposed in [48]. As we can see the semantic completion is required to filter out the clutter as it gives rise to erroneous indentions. We also compared the results quantitatively with the 3D model that was created by the Blender artists (see Figure 15). From this Blender model, a simplified version was created that is watertight. Next, we voxelized the groundtruth mesh as well as the reconstructed mesh as shown in Figure 16. As a result, a binary occupancy grid is obtained in which the points that lie inside the mesh are marked as occupied. Hence, to quantitatively compare the results, we can build a confusion matrix and compute the following two metrics:

- Completeness (red):
  $$100^{-\frac{FN}{TP+FN}}$$
  The completeness metric ranges from 0.01 to 1, with a higher value indicating that a larger proportion of the groundtruth voxelized model is present in the reconstructed model.
- Correctness (blue):
  $$100^{-\frac{FP}{TP+FN}}$$
  The correctness metric ranges from 0, implying an incorrect reconstruction of the entire reconstructed model, to 1, indicating that the reconstructed model contains no redundant voxels. compared to the groundtruth voxelized model.

In Figure 15, the computed metrics are shown for the geometric reconstruction without and with semantic completion. As we can see in Figure 14, the reconstructed rooms with semantic completion no longer have erroneous indentations, which are shown in red, except in the living room. As a result, the completeness metric is 1 for all these rooms as shown in Figure 15. The correctness metric, on the other hand, has slightly dropped for some of the rooms, such as those on the first floor. This is mostly the result of planes that are slightly misaligned. The remaining erroneous indentations and bulges are the result of permanent structures that remained undetected. This can happen either because the completion did not work or the voxels had been given the wrong label or both.

## 5. Conclusions and Future Work

In this paper, we presented a pipeline to extract planar structural components (e.g., walls, ceilings and floors) from a point cloud by first performing semantic scene completion using a deep learning framework. We started from the existing deep learning framework ScanComplete, which was proposed by [11]. However, this framework assumes prior knowledge about the scanning trajectory is available, which is not always the case when using consumer 3D cameras as they are more closed-source. Therefore, we adapted the network to use a different input data representation. We proposed the Manhattan distance based FTDF distance field. Next, we optimized the pipeline in Tensorflow to reduce the overall computation time (up to a factor of 10). The source code for this work is available at our EAVISE website (https://iiw.kuleuven.be/onderzoek/eavise). Furthermore, the original dataset on which the network was trained did not represent our data very well, so we constructed our own dataset.

The results show that our optimized pipeline has a great potential to improve indoor reconstruction results. Furthermore, we performed an ablation study on our design choices and we validated our framework on both synthetic and real data. The framework works very well on synthetic data, but on real data, there is still room for improvement. First of all, more advanced ways of data augmentation can be explored. These include generating multiple synthetic scanning trajectories for each room as a way to increase the amount of training data. Secondly, the rooms in the training and test dataset are now all perfectly axis-aligned. This is, however, not the case in real-life data as small deviations can occur. Hence, we must not only rotate the rooms 90 degrees, but we must also apply smaller perturbations to avoid overfitting to these axis-aligned cases. Finally, the noise and drift on the poses from the scanning trajectory, as well as the noise on the depth data, should be modeled in order for the synthetic data to better resemble the input data.

## Figures and Tables

**Table 1:** Semantic completion results on the SUNCG testset. *Baseline network* shows reference results using our proposed algorithm as described in the previous section, and should be similar to those using the referred ScanComplete network. Differences between the numbers reported here and in [11] are caused by differences in evaluation metric. *Previous groups* represents the network trained using previous voxel group predictions. *Limited dataset* shows the performance drop when using only 1% of the data for training, indicating the potential gain when adding more data. Classes are indicated in the rightmost columns: bed, ceiling (ceil.), chair, floor, furniture (furn.), object (obj.), sofa, desk, tv, wall, window (wind.), and the class average (avg.). *Combined classes* illustrates the performance increase when combining multiple classes under a single label.

	Scene Completion			Semantic Scene Completion											
**Method**	**Prec.**	**Recall**	**IoU**	**Bed**	**Ceil.**	**Chair**	**Floor**	**Furn.**	**Obj.**	**Sofa**	**Desk**	**Tv**	**Wall**	**Wind.**	**Avg.**
Baseline network [11]	85.69	65.99	59.56	22.95	58.46	14.91	71.99	16.95	27.63	28.17	22.32	6.29	63.77	6.41	30.90
Previous groups	85.10	66.04	59.20	13.47	55.82	24.53	71.92	34.13	36.17	43.17	43.61	15.78	69.23	6.08	37.63
Limited dataset	82.52	66.34	58.17	20.38	58.19	10.42	69.43	15.57	15.74	20.43	18.04	0.25	58.78	5.47	26.61
Combined classes	88.41	94.03	**83.71**	-	84.07	-	85.07	-	44.97	-	-	-	68.94	11.13	**58.84**

**Table 2:** Semantic completion results on our testset, with classes floor, ceiling (ceil.), slanted ceiling (sl. ceil.), window (wind.), door frame (dr. fr.), wall, door, object (obj.), and average (avg.). *Prep.* refers to the preprocessing strategy (padded or cropped), *augm.* refers to whether or not data augmentation was applied and *enc.* refers to the input encoding (TSDF [11] or our proposed distance field FTDF).

Method			Scene Completion			Semantic Scene Completion								
**Prep.**	**Augm.**	**Enc.**	**Prec.**	**Recall**	**IoU**	**Floor**	**Ceil.**	**Sl. Ceil.**	**Wind.**	**Dr. Fr.**	**Wall**	**Door**	**Obj.**	**Avg.**
crop	no	ftdf [ours]	98.04	98.82	96.91	97.84	83.55	42.07	0.00	0.00	42.09	3.47	32.06	37.63
pad	no	ftdf [ours]	99.55	99.38	98.93	98.77	**93.32**	**69.44**	45.38	47.70	77.62	26.73	57.32	64.54
pad	no	tsdf [11]	98.51	98.33	96.89	96.68	71.25	41.90	41.85	54.11	11.68	13.43	31.25	45.27
pad	yes	ftdf [ours]	99.61	99.53	**99.14**	**98.84**	92.99	68.57	53.90	**48.82**	**84.55**	**45.63**	**62.85**	**69.52**
